# Berberine stimulates lysosomal AMPK independent of PEN2 and maintains cellular AMPK activity through inhibiting the dephosphorylation regulator UHRF1

**DOI:** 10.3389/fphar.2023.1148611

**Published:** 2023-04-18

**Authors:** Gang Ren, Yu-Wei Ding, Lu-Lu Wang, Jian-Dong Jiang

**Affiliations:** ^1^ Department of Virology, Institute of Medicinal Biotechnology, Chinese Academy of Medical Sciences and Peking Union Medical College, Beijing, China; ^2^ Diabetes Research Center, Chinese Academy of Medical Sciences and Peking Union Medical College, Beijing, China; ^3^ State Key Laboratory of Bioactive Substance and Function of Natural Medicines, Institute of Materia Medica, Chinese Academy of Medical Sciences and Peking Union Medical College, Beijing, China

**Keywords:** AMPK, berberine, metformin, AXIN1, UHRF1

## Abstract

**Aim:** AMPK is the key regulatory kinase mediating the effect of berberine (BBR) and metformin on metabolic improvement. The present study investigated the mechanism of BBR on AMPK activation at low doses, which was different from that of metformin.

**Methods:** Lysosomes were isolated, and AMPK activity assay was performed. PEN2, AXIN1 and UHRF1 were investigated through gain/loss of function approaches, including overexpression, RNA interfering and CRISPR/Cas9-mediated gene knockout. Immunoprecipitation was utilized for detecting the interaction of UHRF1 and AMPKα1 after BBR treatment.

**Results:** BBR activated lysosomal AMPK, but weaker than metformin. AXIN1 mediated BBR’s effect on lysosomal AMPK activation, while PEN2 did not. BBR, but not metformin, decreased UHRF1 expression by promoting its degradation. BBR reduced the interaction between UHRF1 and AMPKα1. And overexpression of UHRF1 abolished the effect of BBR on AMPK activation.

**Conclusion:** BBR activated lysosomal AMPK as dependent on AXIN1, but not PEN2. BBR maintained cellular AMPK activity by reducing UHRF1 expression and its interaction with AMPKα1. The mode of action of BBR was different from that of metformin on AMPK activation.

## Introduction

Berberine (BBR) and metformin possess similar pharmacological activities with aspect to hypoglycemic/hypolipidemic effects, anti-inflammation, and anti-cancer, although the molecular formulas of them are different (Kong et al., 2020; Lv and Guo, 2020). The mechanism studies show that AMP-activated protein kinase (AMPK) is the key regulatory protein mediating the action of BBR and metformin (Zhou et al., 2001; Lee et al., 2006). Both BBR and metformin activated AMPK indirectly by inhibiting respiratory complex I and elevating cellular AMP/ATP (Turner et al., 2008; Bridges et al., 2023).

However, recent reports suggested that clinically relevant doses of metformin did not increase the AMP/ATP or ADP/ATP ratio, but activated AMPK through presenilin enhancer, gamma-secretase subunit (PSENEN and PEN2), and AXIN1 of lysosomal pathway (Hawley et al., 2002; Zhang et al., 2016; Ma et al., 2022). Also, it has been reported that BBR activated AMPK but significantly reduced AMP/ATP ratio in the livers of fructose-fed mice (Li et al., 2020). This phenomenon indicates that BBR could activate AMPK through other mechanisms at clinically relevant doses. Consistent with this, our previous study has shown that BBR activated cellular AMPK at the low doses of 0.1–2.5 μM, which was more clinically relevant (Ren et al., 2020). Therefore, we investigated whether BBR activated AMPK through a lysosomal pathway similar to low doses of metformin.

On the other hand, AMPK activity is negatively regulated through dephosphorylation. A recent study reported that ubiquitin-like with plant homeodomain and RING finger domains 1 (UHRF1) promoted protein phosphatase 2A (PP2A)-dependent dephosphorylation of AMPKα through the UHRF1-PPP2CA (protein phosphatase 2 catalytic subunit alpha, the catalytic subunit of PP2A) complex and hence suppressed cellular AMPK activity (Xu et al., 2022). Also, a previous study showed that BBR reduced UHRF1 expression in multiple myeloma cells (Gu et al., 2020). Therefore, we investigated whether BBR activated AMPK through downregulation of UHRF1 in the present study.

## Materials and methods

### Reagents and kits

Dimethyl sulfoxide (DMSO, D2438) and metformin (D150959) were purchased from Sigma-Aldrich Co. LLC (St. Louis, MO, United States of America). Berberine (BBR, HY-18258), MK-8722 (HY-111363), AICAR (Acadesine, 5-aminoimidazole-4-carboxamide ribofuranoside, HY-13417), cycloheximide (CHX, HY-12320), and MG-132 (HY-13259) were purchased from MedChemExpress Co. LLC (Princeton, NJ, United States of America). Fetal bovine serum (FBS, #10091148), Iscove’s modified Dulbecco’s medium (IMDM, #12440053), 0.25% trypsin-EDTA (#25200072), phosphate-buffered saline (PBS, #20012027), Lipofectamine™ 3,000 transfection kit (L3000015), protease/phosphatase inhibitors (#78442), puromycin dihydrochloride (A1113803), Opti-MEM™ I reduced serum medium (#31985070), cellular protein extraction kit (#78501), BCA protein quantification kit (A53225) and ATP Determination Kit (A22066) were purchased from Thermo Fisher Scientific Inc. (Shanghai, China). AMP-Glo™ Assay kit (V5011) was purchased from Promega Biotech Co., Ltd. (Beijing, China). The rabbit and mouse antibodies against AMPKα (#2793), p-AMPKα (Thr172) (#2535), ACC (#3676), p-ACC (Ser79) (#3661), Raptor (#2280), p-Raptor (Ser792) (#2083), ULK1 (#8054), p-ULK1 (Ser555) (#5869), AXIN1 (#2087), Myc-tag (#2276), *ß*-actin (#4970), anti-rabbit IgG HRP-linked antibody (#7074) and anti-mouse IgG HRP-linked antibody (#7076) were purchased from Cell Signaling Technology, Inc. (Danvers, MA, United States of America). The antibodies against PEN2 (ab154830) and UHRF1 (ab213223) were purchased from Abcam (Cambridge, MA, United States of America). The antibodies against PPP2CA (13482-1-AP) and DYKDDDDK tag (binds to FLAG^®^ tag epitope) (20543-1-AP) were purchased from Proteintech Group, Inc. (Chicago, IL, United States of America).

The AXIN1 CRISPR/Cas9 knockout (KO) plasmid (sc-401074), AXIN1 homology-directed repair (HDR) plasmid (sc-401074-HDR) and Protein A/G PLUS-Agarose beads (sc-2003) were purchased from Santa Cruz Biotechnology, Inc. (Dallas, TX, United States of America). The plasmids of UHRF1 cDNA open reading frame (ORF) clone (Human, untagged, HG17896-UT), PPP2CA cDNA ORF clone (Human, C-GFPSpark^®^ tag, HG10420-ACG), pCMV3-untagged negative control vector (CV011), UHRF1 cDNA ORF clone (Human, C-DYKDDDDK Flag^®^ tag, HG17896-CF), AMPK alpha 1/PRKAA1 cDNA ORF clone (Human, C-Myc tag HG11488-CM), pCMV3-C-FLAG negative control vector (C-terminal FLAG-tagged, CV012) and pCMV3-C-Myc negative control vector (C-terminal Myc-tagged, CV014) were purchased from Sino Biological Inc. (Beijing, China). The Minute^TM^ Lysosome Isolation Kit for mammalian cells/tissues (LY-034) was purchased from Invent Biotechnologies Inc. (Beijing, China). The Cyclex^®^ AMPK Kinase Assay Kit (CY-1182) was purchased from Cyclex Co. Ltd (Nagano, Japan). The cellular RNA extraction kit (#74106) was purchased from Qiagen Co. Ltd. (Shanghai, China). Kits for reverse transcription (RT, RR037A) and real-time RT-PCR (RR820A) were purchased from Takara Bio Inc. (Shiga, Japan). The immunoprecipitation (IP) lysis buffer (C1054) was purchased from Applygen Technologies Inc. (Beijing, China).

### Cell culture, transfection and drug treatment

The human colon cancer HCT-116 cell line was originally obtained from the Cell Culture Center of Peking Union Medical College (Beijing, China), and cultured in IMDM plus 10% FBS at 37°C in a 5% CO_2_ incubator.

For AXIN1 KO, HCT-116 cells were co-transfected with AXIN1 CRISPR/Cas9 KO and HDR plasmids, and stable AXIN1^−/−^ transfectants were selected with 2 μg/mL puromycin and confirmed by visualization of green fluorescence, as described previously (Ren et al., 2020). After overnight starvation and drug treatment, the cells were lysed for Western blot and AMPK activity assay.

For UHRF1 and PPP2CA overexpression, HCT-116 cells were cultured in 12-well plates at a density of 6 × 10^5^/well, and transiently transfected with 1.25 μg of the pCMV3-untagged negative control plasmid, UHRF1 cDNA ORF clone plasmid, and PPP2CA cDNA ORF clone plasmid, respectively. After drug treatment, the cells were lysed for Western blot and AMPK activity assay.

### RNA interference

For siRNA knockdown of AXIN1, PEN2 and UHRF1 expression, HCT-116 cells were cultured in 12-well plates and transfected with 40 pmol of the corresponding siRNAs. After drug treatment, the cells were lysed for Western blot and AMPK activity assay. The siRNA sequences are listed in Table 1.

**TABLE 1:** Sequences of siRNAs used in human HCT-116 cells.

Genes	Sense	Anti-sense
NC	UUC​UCC​GAA​CGU​GUC​ACG​UTT	ACG​UGA​CAC​GUU​CGG​AGA​ATT
AXIN1	CCG​AAA​GUA​CAU​UCU​UGA​UAA​TT	UUA​UCA​AGA​AUG​UAC​UUU​CGG​TT
PEN2	GAU​CAC​CAU​CUU​CCA​GAU​CUA​TT	UAG​AUC​UGG​AAG​AUG​GUG​AUC​TT
UHRF1	CGU​CAU​UUA​CCA​CGU​GAA​AUA​TT	UAU​UUC​ACG​UGG​UAA​AUG​ACG​TT

### Lysosome isolation

After drug treatment, HCT-116 cells were collected for lysosome isolation using a commercial kit with a spin-column based technology. Briefly, cells were lysed after passing through the filter cartridge by centrifugation at 16,000 g for 30 s at 4°C. Lysosomes were isolated from the nucleus, mitochondria, cell debirs and other organelles after three runs of centrifugation and lysed in the corresponding reagents for Western blot and AMPK activity assay.

### Determination of intracellular AMP and ATP

Both AMP and ATP were detected by commercial kits on the basis of bioluminescence assay, as described previously (Li et al., 2023). After BBR treatment, cells were lysed and reaction was started with prepared reagents according to the manufactures’ protocols. The luminescence was measured with a plate-reading luminometer, and the AMP/ATP ratio was calculated.

### Western blot

After drug treatment, total proteins extracted from cells/lysosomes were quantified using a BCA kit. An equivalent of 20 μg was separated by SDS-PAGE and transferred onto PVDF membranes. Then, the membranes were probed with specific antibodies to detect the target proteins. The immunoreactive bands were visualized using *ß*-actin as an internal control.

### AMPK activity assay

AMPK activity was determined using a commercial ELISA kit, as described previously (Ren et al., 2020). Briefly, cell lysates were transferred to a plate pre-coated with a substrate peptide of AMPK corresponding to mouse insulin receptor substrate 1 (IRS-1). An anti-phospho-mouse IRS-1 S789 monoclonal antibody was employed to measure the amount of phosphorylated substrate. The “relative AMPK activity” was presented as a fold-change of the vehicle-treated group after subtracting the OD450 readings of Compound C (inhibitor control)-treated parallel samples.

### Real-time RT-PCR

Total RNA was extracted and reversely transcribed into cDNA, and quantitative RT-PCR was performed by SYBR Green method using commercial kits. The relative levels of target genes were represented as fold of the vehicle-treated control group. The primers are listed in Table 2.

**TABLE 2:** Primers for real-time PCR (5′to 3′).

Genes	Sense primer	Anti-sense primer
UHRF1	GGC​AAG​TGG​AAG​CGG​AAG​TCG	CTT​GGC​GTT​GCT​CTT​GTC​CTC​TC
β-actin	TCA​ACA​CCC​CAG​CCA​TGT​A	AGT​ACG​GCC​AGA​GGT​GTA​CG

### IP

HCT-116 cells were cultured in 10-cm dish at a density of 1.5 × 10^7^/dish and co-transfected with 14 μg of the plasmids encoding Myc-AMPKα1 and FLAG-UHRF1 or the corresponding empty vectors (pCMV3-C-FLAG negative control vector and pCMV3-C-Myc negative control vector) at a ratio of 1:1. After 24 h, one dish of cells was starved overnight and treated with BBR and DMSO (vehicle) for 1 h, respectively. Cellular total proteins were extracted using 1 mL IP lysis buffer. After centrifugation at 12,000 g for 30 min, the lysate was incubated with the anti-FLAG antibody overnight. Protein A/G beads were equilibrated with PBS buffer, and then added into the lysate and antibody mixture. After rotated for 3 h at 4°C and washed, the beads were mixed with 2 × SDS loading buffer and boiled 10 min for Western blot of anti-FLAG and anti-Myc antibodies.

### Statistical analysis

The data were analyzed by one-way and two-way analysis of variance (ANOVA), respectively, depending on the data types; *p* < 0.05 indicated statistical significance.

## Results

### BBR activated lysosomal AMPK moderately at low doses

**FIGURE 1:**
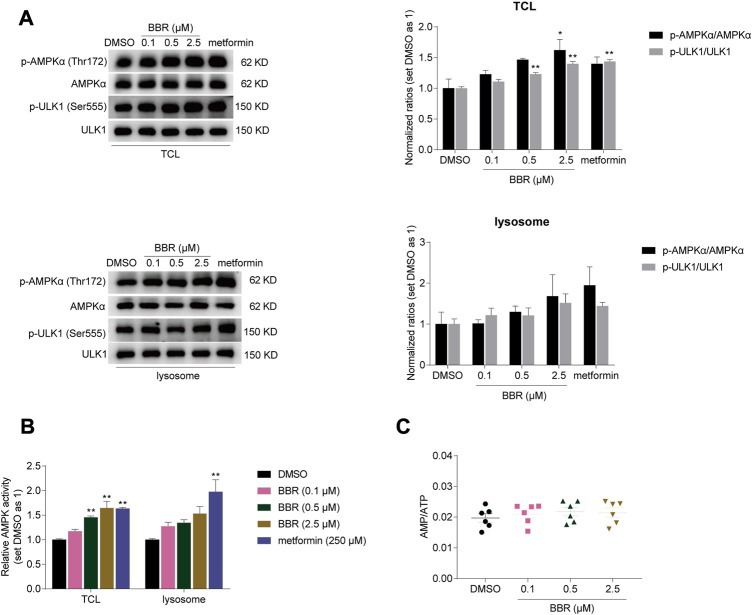
BBR activated lysosomal AMPK at the low doses. BBR increased phosphorylation of AMPKα and ULK1 **(A)**, and simulated AMPK activity **(B)** in total cell lysates (TCL) and lysosomes of HCT-116 cells. BBR did not increase cellular AMP/ATP ratio at low doses **(C)**. Cells were treated with DMSO, BBR (0.1, 0.5, 2.5 μM) and metformin (250 μM) for 20 h. The ratio of DMSO treated group was set as 1 in TCL and lysosomes, respectively **(A, B)**. Values are expressed as the mean ± SEM of 3-6 independent experiments; *p < 0.05, **p < 0.01 *versus* that of the corresponding vehicle (DMSO) treated group.

Firstly, we detected whether BBR activated lysosomal AMPK. The results showed that BBR increased the phosphorylation of AMPKα (Thr172) and its downstream target ULK1 (Ser555) (Egan et al., 2011) in both total cell lysates (TCL) and isolated lysosomes of HCT-116 cells, similar to that of metformin (Figure 1A).

Consistent with this, AMPK activity was increased by 17%–65% and 28%–54% by BBR treatment at 0.1–2.5 μM in TCL and lysosomes respectively, as detected by ELISA for quantitative detection (Figure 1B). Metformin increased the AMPK activity by 63% and 97% at 250 μM in TCL and lysosomes. It seems that metfromin was stronger than BBR in activating lysosomal AMPK at clinically relevant doses.

In the meanwhile, BBR did not increase the AMP/ATP ratio at the dose of 0.1–2.5 μM in HCT-116 cells (Figure 1C). These results indicated that BBR activated cellular and lysosomal AMPK without raising AMP/ATP ratio at low doses, which was consistent with the previous reports (Lv et al., 2012; Li et al., 2020).

### AXIN1 but not PEN2 was crucial for BBR-induced AMPK activation

Next, we investigated whether the key regulatory proteins in the lysosomal pathway were crucial for BBR on AMPK activation. First, we detected PEN2, as it was indispensable for metformin-induced lysosomal AMPK activation at low doses (Ma et al., 2022).

**FIGURE 2:**
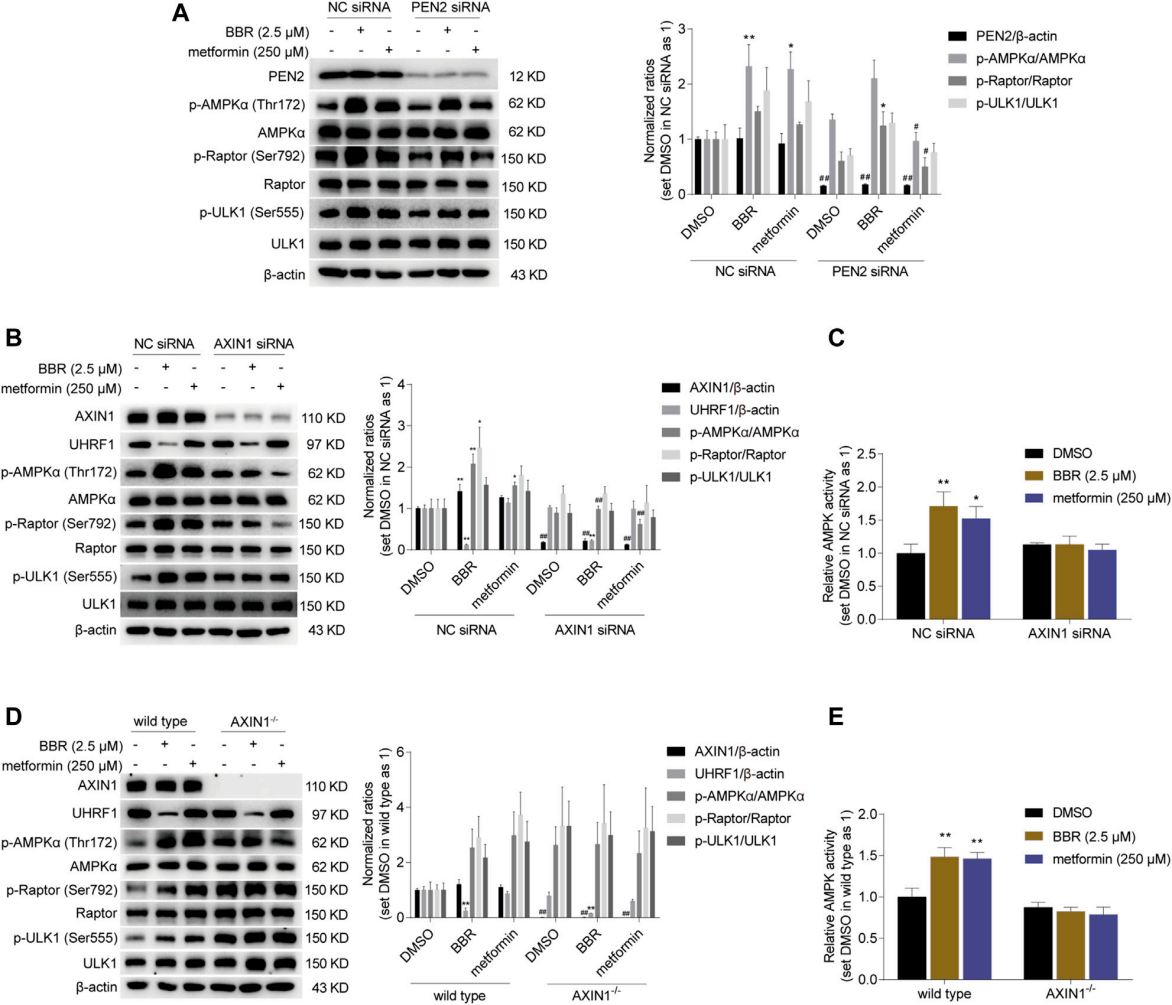
BBR induced AMPK activation was diminished in AXIN1 knockdown and AXIN1^−/−^ cells. BBR’s effect was not influenced on phosphorylation of AMPKα, Raptor and ULK1 in PEN2 knockdown cells **(A)**. BBR’s effect was decreased on stimulating AMPK activity in AXIN1 knockdown cells **(B, C)**. HCT-116 cells were transfected separately with PEN2, AXIN1 and non-sense control (NC) siRNAs. 24 h after transfection, cells were treated with DMSO, BBR (2.5 μM) and metformin (250 μM) for 20 h, respectively. BBR’s effect was inhibited on promoting AMPK activation in AXIN1^−/−^ cells **(D, E)**. AXIN1 was knocked out in HCT-116 cells and the monoclonal transfectants were selected as described in Methods. The wild type and AXIN1^−/−^ cells were starved overnight and treated with DMSO, BBR (2.5 μM) and metformin (250 μM) for 20 h, respectively. The ratio was set as 1 in the NC siRNA transfected **(A–C)** and wild type **(D, E)** cells treated with DMSO, respectively. Data are expressed as the mean ± SEM of three separate experiments; ^*^ *p* < 0.05, ^**^ *p* < 0.01 *versus* that of the vehicle (DMSO) treated group in the same transfected cells; ^#^ *p* < 0.05, ^##^ *p* < 0.01 *versus* that of the corresponding treated group in the NC siRNA transfected or wild type cells.

Interestingly, small interfering (si) RNA knockdown of PEN2 significantly inhibited the phosphorylation of AMPKα and Raptor induced by metformin, but not BBR. As shown in Figure 2A, BBR and metformin increased the phosphorylation level of AMPKα and its downstream target ULK1 and Raptor (Gwinn et al., 2008) in non-sense control (NC) siRNA transfected cells. However, the effect of metformin was blunted after PEN2 downregulation by siRNA, while BBR’s action was not affected on phosphorylation of AMPKα, Raptor and ULK1 by PEN2 knockdown (Figure 2A).

Then, we focused on AXIN1, which played an essential role in co-translocating LKB1 onto the v-ATPase-Ragulator complex for lysosomal AMPK activation (Zhang et al., 2016). As shown in Figure 2B, RNA interference of AXIN1 significantly reduced AXIN1 expression, and the effect of BBR and metformin on the phosphorylation of AMPKα, Raptor and ULK1 was abolished. Consistent with this, BBR and metformin stimulated AMPK activation in the scrambled non-sense control (NC) siRNA transfected HCT-116 cells, while the effect was significantly reduced in the cells transfected with AXIN1 siRNA, as detected by ELISA (Figure 2C).

Finally, to confirm the role of AXIN1, we performed KO in HCT-116 cells. The results showed that the effect of BBR and metformin was inhibited on promoting phosphorylation of AMPKα, Raptor and ULK1 in AXIN1^−/−^ HCT-116 cells, compared to that in wild type cells (Figure 2D). Consistent with this phenomenon, the effect of BBR and metformin was diminished on AMPK activation in AXIN1^−/−^ cells, as revealed by ELISA (Figure 2E).

These results suggested that AXIN1, but not PEN2, was crucial for BBR to stimulate AMPK activation. This finding indicated that the mode of action of BBR might be different from that of metformin on cellular AMPK activation.

### BBR was an AMPK activator reducing UHRF1 expression, differing from metformin, AICAR and MK-8722

**FIGURE 3:**
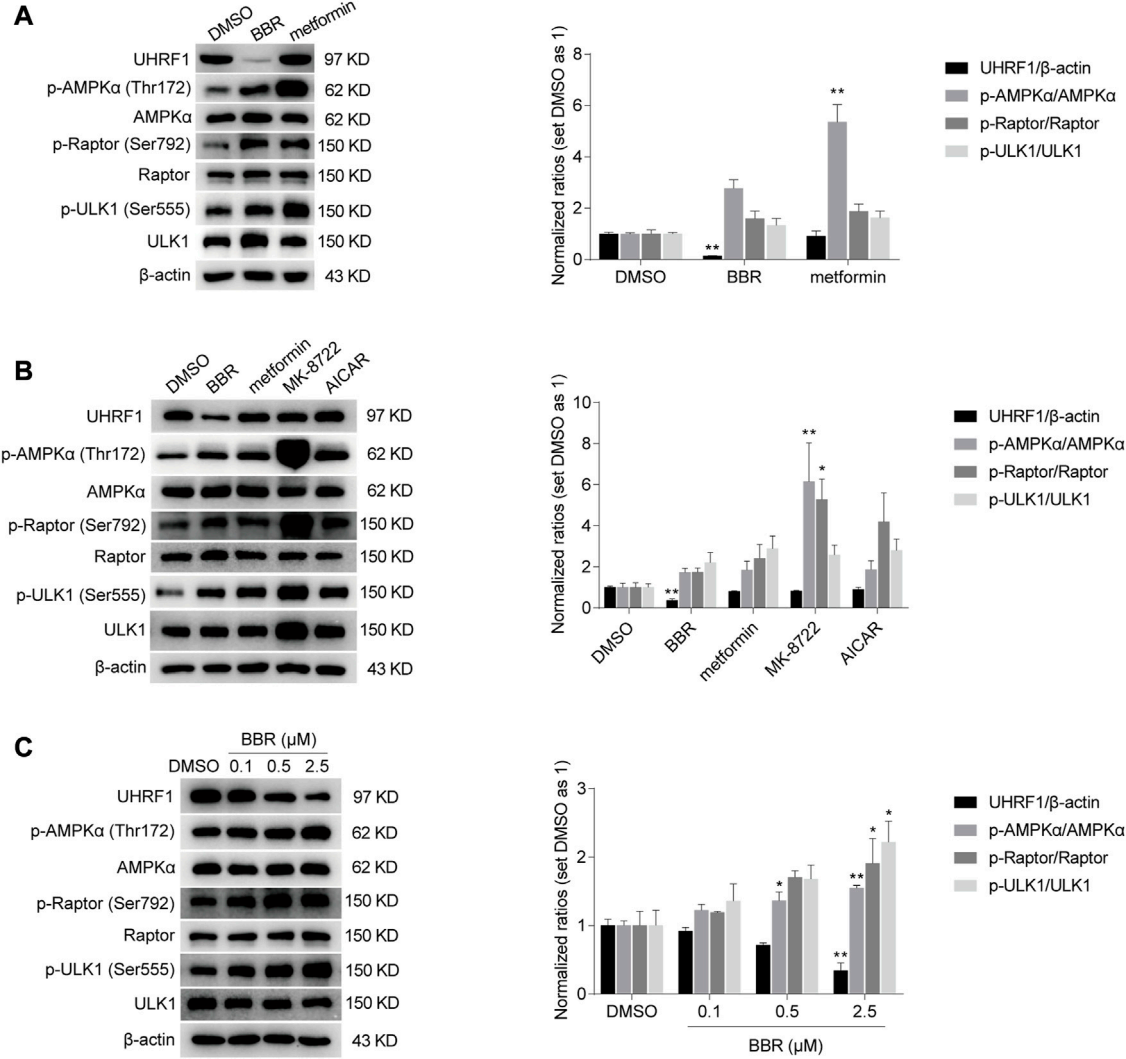
BBR, but not other AMPK activators such as metformin, MK-8722 and AICAR, reduced UHRF1 expression. BBR decreased UHRF1 expression, but metformin did not even at a high dose of 2 mM **(A)**. The direct activators MK-8722 and AICAR did not influence UHRF1 expression **(B)**. BBR downregulated UHRF1 in a dose-dependent manner **(C)**. HCT-116 cells were treated with DMSO, BBR (2.5 μM in panel A and B; 0.1 μM, 0.5 μM and 2.5 μM in panel **(C)**, metformin (2 mM in panel A; 250 μM in panel **(B)**, MK-8722 (10 μM, panel **(B)** and AICAR (500 μM, panel **(B)** for 20 h, respectively. The ratio of DMSO treated group was set as 1. Values are expressed as the mean ± SEM of three independent experiments; ^*^ *p* < 0.05, ^**^ *p* < 0.01 *versus* that of the vehicle (DMSO) treated group.

As described in Introduction, AMPK activity is negatively regulated by UHRF1 through dephosphorylation of AMPKα. We hypothesized that BBR and metformin inhibited UHRF1 expression to sustain the phosphorylation of AMPKα and promote AMPK activity. Firstly, we investigated whether the expression of UHRF1 was downregulated by BBR or metformin. As shown in Figure 3A, BBR significantly decreased the UHRF1 protein expression at a dose of 2.5 μM, while metformin did not influence UHRF1 expression at the high dose of 2 mM. These results led us to investigate other AMPK activators, such as 5-aminoimidazole-4-carboxamide ribofuranoside (AICAR) and MK-8722.

AICAR is 5′-phosphorylated and converted to its active form ZMP to mimic the action of AMP as a nucleoside analog in cells. MK-8722 is a direct pan-AMPK activator that acts through interaction with the allosteric drug and metabolite (ADaM) site located between AMPK *a* and *ß* subunits (Myers et al., 2017; Rhein et al., 2021). The results showed that only BBR downregulated UHRF1 expression among these AMPK activators, although all these agents activated AMPK, as revealed by the phosphorylation level of AMPKα, Raptor and ULK1 (Figure 3B).

In addition, BBR reduced UHRF1 expression in a dose dependent manner. In contrast, the phosphorylation levels were increased significantly on AMPKα, Raptor and ULK1 by BBR treatment (Figure 3C). These results suggested that BBR might activate AMPK by downregulating UHRF1 expression.

### BBR downregulated UHRF1 expression by promoting its degradation

**FIGURE 4:**
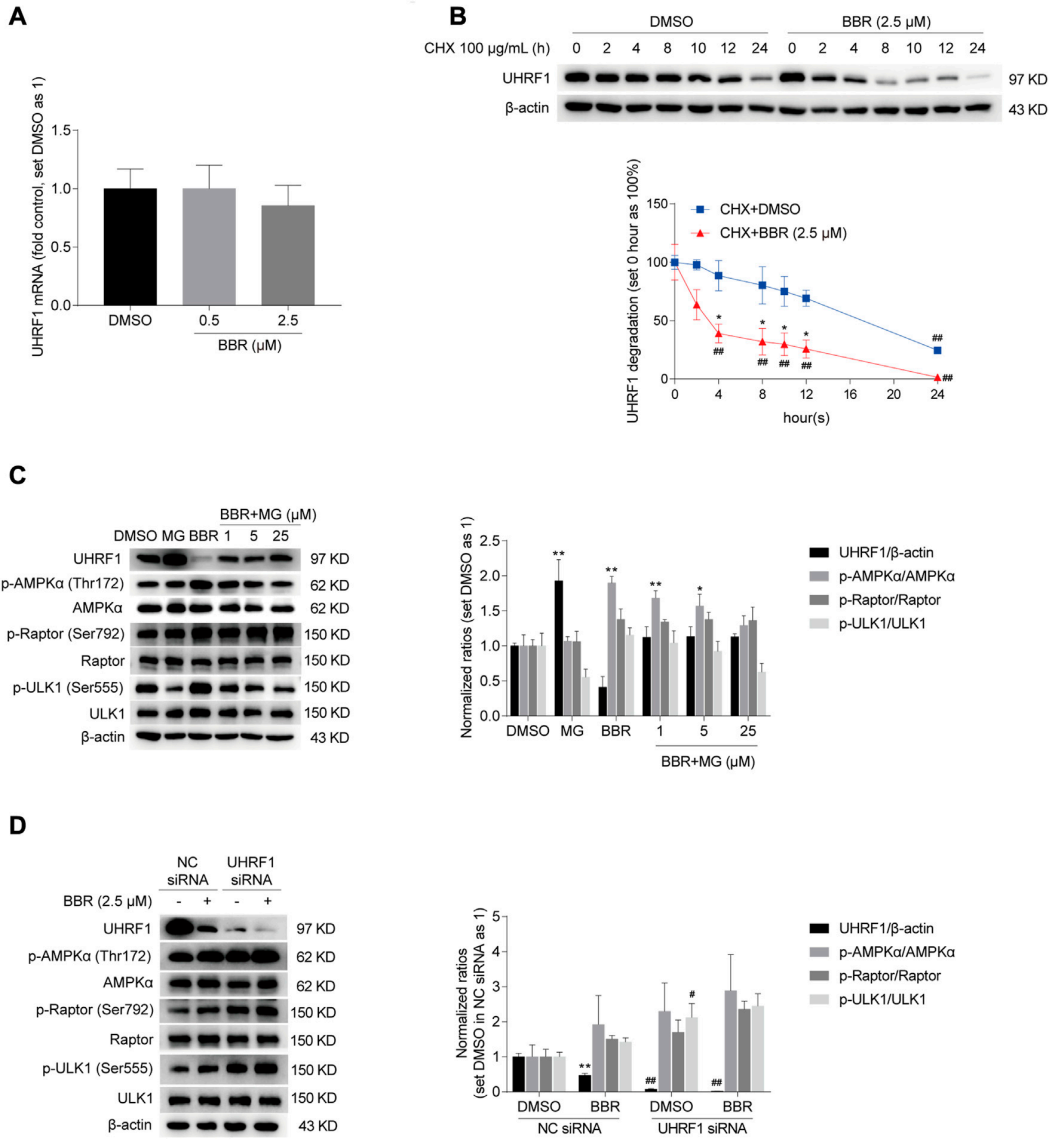
BBR downregulated UHRF1 by promoting its degradation. BBR hardly decreased the mRNA expression of UHRF1 **(A)**. HCT-116 cells were treated with BBR (0.5 and 2.5 μM) and DMSO for 20 h, respectively. UHRF1 degradation was accelerated by BBR treatment **(B)**. HCT-116 cells were treated with 100 μg/mL CHX, combined with DMSO and BBR (2.5 μM) for 0, 2, 4, 8, 10, 12 and 24 h, respectively. The ratio of 0 h was set as 100% in DMSO and BBR treated groups, respectively. MG-132 abolished BBR’s effect on UHRF1 downregulation and AMPK activation **(C)**. HCT-116 cells were treated with DMSO or BBR (2.5 μM) alone for 24 h, or pretreatment with DMSO or BBR (2.5 μM) for 20 h and combined with MG-132 (MG, 25 μM; BBR + MG, 1 μM, 5 μM and 25 μM) for an additional 4 h. The ratio of DMSO-treated group was set as 1. BBR further reduced UHRF1 expression in UHRF1 siRNA transfected cells **(D)**. HCT-116 cells were transfected independently with UHRF1 and NC siRNAs. 48 h post-transfection, cells were treated with DMSO and BBR (2.5 μM) for 20 h, respectively. The ratio was set as 1 in the DMSO group of NC siRNA transfected cells. Values are presented as the mean ± SEM of three independent experiments; ^*^ *p* < 0.05, ^**^ *p* < 0.01 *versus* that of the corresponding DMSO or CHX + DMSO treated group; ^#^ *p* < 0.05, ^##^ *p* < 0.01 *versus* that of the corresponding “0 h” group treated with DMSO and BBR, respectively **(B)**, or that of the corresponding treated group in the NC siRNA transfected cells **(D)**.

To prove this, we first investigated the mechanism on BBR-mediated downregulation of UHRF1. As shown in Figure 4A, BBR decreased the UHRF1 mRNA expression by only 14% at the dose of 2.5 μM, compared to the vehicle (DMSO) treated group in HCT116 cells. And at the lower dose of 0.5 μM, BBR hardly reduced UHRF1 mRNA. It seems not sufficient to explain the significant downregulation of its protein level at the same doses (Figure 3C). Hence, we focused on the protein degradation process of UHRF1.

As shown in Figure 4B, BBR significantly decreased UHRF1 expression 4 h after co-treatment with CHX in HCT-116 cells, compared to the group treated with DMSO and CHX. And the effect of BBR on promoting UHRF1 degradation lasted for 24 h. Moreover, the effect of BBR on UHRF1 downregulation and AMPK activation was restrained by the proteasome inhibitor MG-132. Specifically, BBR promoted phosphorylation of AMPKα, Raptor and ULK1, but the effect was inhibited after co-treatment with MG-132 in HCT-116 cells. Also, UHRF1 expression was restored in the BBR and MG-132 co-treatment groups compared to the level in the DMSO and BBR treatment groups, respectively. Actually, UHRF1 expression was increased significantly by MG-132 alone at the dose of 25 μM.

Finally, as shown in Figure 4D, UHRF1 siRNA significantly decreased UHRF1 expression in HCT-116 cells. And the phosphorylation level was elevated on AMPKα, ULK1 and Raptor after UHRF1 knockdown. Further, BBR downregulated UHRF1 expression and increased the phosphorylation of AMPKα, Raptor and ULK1 in UHRF1 siRNA transfected cells. It is indicated that UHRF1 expression was knocked down by siRNAs at the mRNA level and further downregulated after BBR treatment at the protein level. Accordingly, p-AMPKα level was increased with UHRF1 siRNA transfection and further stimulated by BBR.

These results suggested that BBR downregulated UHRF1 expression by promoting its protein degradation, especially through the proteasome pathway. It is also indicated that BBR might stimulate AMPK through downregulation of UHRF1.

### Overexpression of UHRF1 abolished BBR’s effect on AMPK activation

**FIGURE 5:**
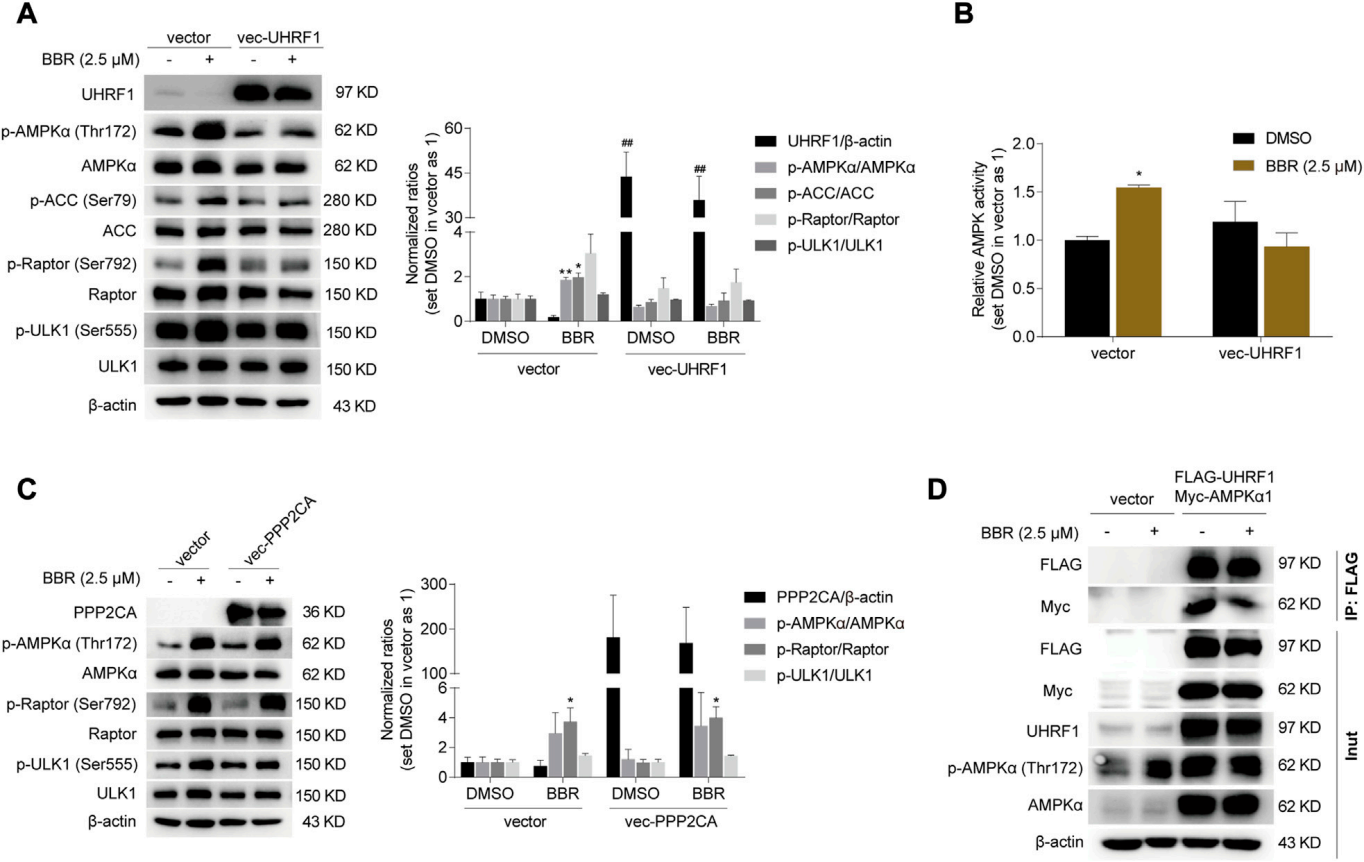
UHRF1 overexpression abolished the effect of BBR on AMPK activation. Overexpression of UHRF1 abolished BBR’s effect on stimulating phosphorylation of AMPKα and AMPK activity **(A, B)**. PPP2CA overexpression did not influence BBR’s effect on phosphorylation of AMPKα, Raptor and ULK1 **(C)**. 24 h after transfection, cells were treated with DMSO and BBR (2.5 μM) for 20 h, respectively. The ratio was set as 1 in the empty vector transfected cells treated with DMSO. ^*^ *p* < 0.05, ^**^ *p* < 0.01 *versus* that of the DMSO treated group in the corresponding transfected cells; ^##^ *p* < 0.01 *versus* that of the corresponding treated group in the empty vector transfected cells. BBR reduced the interaction between UHRF1 and AMPKα1 **(D)**. 24 h after transfection, the cells were starved overnight and treated with DMSO and BBR (2.5 μM) for 1 h, respectively. IP was performed as described in Methods.

As described above, AMPK was activated after UHRF1 downregulation by siRNA and BBR (Figure 4D). Hence, we speculated that BBR would be ineffective on AMPK activation after overexpression of UHRF1. As expected, overexpression of UHRF1 significantly attenuated the effect of BBR on AMPK activation (Figure 5A). Specifically, BBR increased the phosphorylation of AMPKα and its downstream proteins ACC, Raptor and ULK1 in empty vector-transfected cells; however, the effect was blunted after transient transfection of the UHRF1-expressing plasmid in HCT-116 cells (Figure 5A). Consistent with this, BBR’s effect on activating AMPK was diminished after UHRF1 overexpression, as revealed by ELISA (Figure 5B).

It is reported that AMPKα was dephosphorylated by the UHRF1-PPP2CA complex (Xu et al., 2022). As shown in Figure 5C, PPP2CA overexpression did not influence the effect of BBR on stimulating the phosphorylation of AMPKα and its downstream targets Raptor and ULK1. This result indicated that UHRF1 but not PPP2CA was the target of BBR on AMPK regulation. And it is speculated that BBR might interfere with the dephosphorylation complex UHRF1-PPP2CA to bind with AMPKα, as BBR is reported to bind with UHRF1 previously (Gu et al., 2020).

Indeed, the interaction between UHRF1 and AMPKα1 was weakened after short time treatment of 1 h with BBR, as revealed by the reduced Myc-AMPKα1 level after co-expression and immunoprecipitation of FLAG-UHRF1 and Myc-AMPKα1 in HCT-116 cells (Figure 5D). It is worth mentioning that BBR significantly decreased UHRF1 expression 4–6 h after cell treatment (Figure 4B, Supplementary Figure S1). Hence, 1 h was an appropriate time point for investigating the interaction between UHRF1 and AMPKα1.

In addition, BBR promoted phosphorylation of AMPKα in the empty vector transfected cells, but the effect was abolished in the cells expressing FLAG-UHRF1 (Figure 5D), which was consistent with the results in Figures 5A,B. And it is indicated that BBR’s action on AMPK activation was eliminated by UHRF1 overexpression.

**FIGURE 6:**
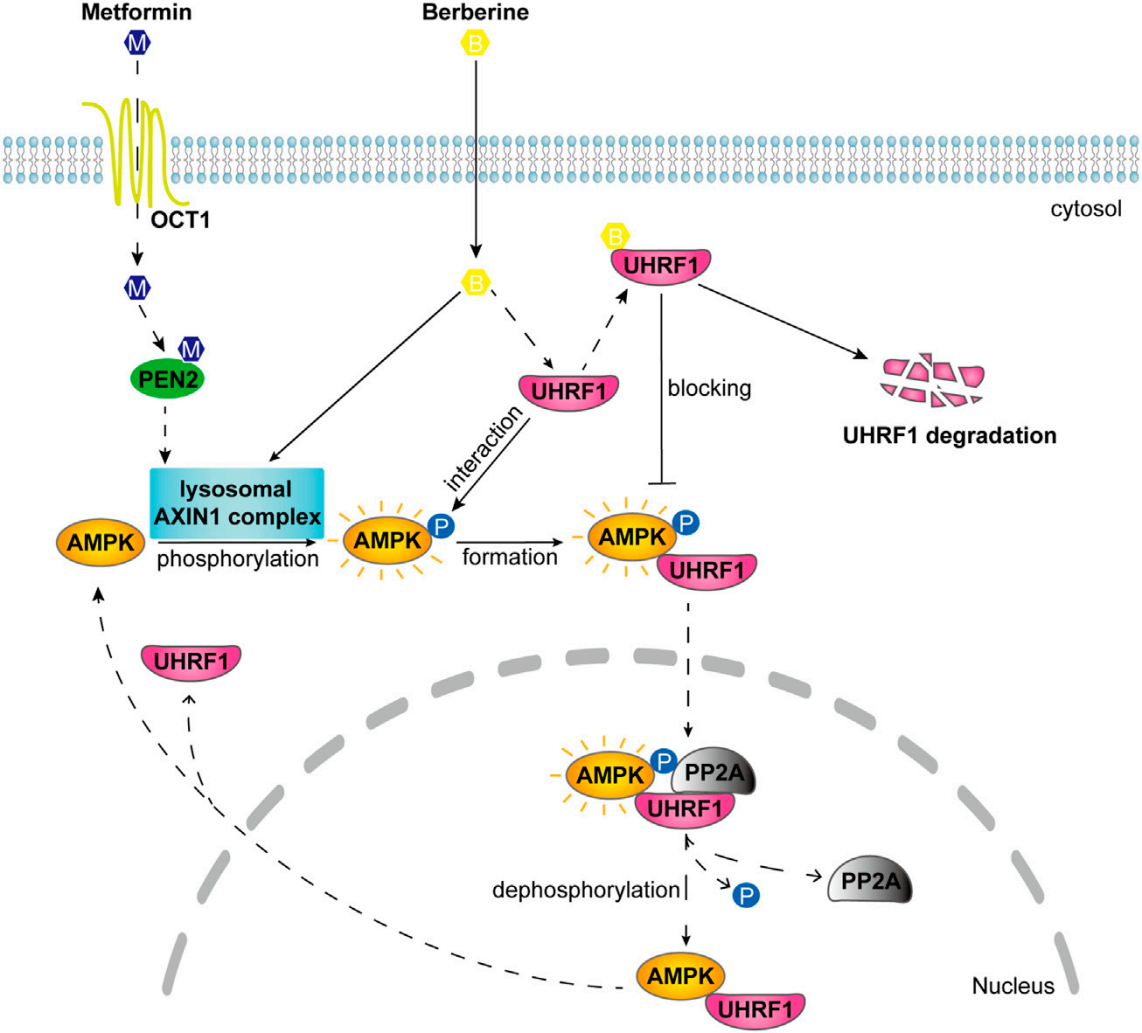
Schematic diagram illustrates the different mode of action on AMPK activation between BBR and metformin. Metformin activates lysosomal AMPK through PEN2 and AXIN1 after transport into the cellular cytosol (Zhang et al., 2016; Ma et al., 2022). BBR activated lysosomal AMPK through AXIN1 but not PEN2. After interaction with AMPKα, UHRF1 recruits PP2A to dephosphorylate AMPK in the nucleus (Xu et al., 2022). And BBR is reported to bind with UHRF1 (Gu et al., 2020). BBR inhibited the dephosphorylation process of AMPK by reducing the interaction of UHRF1 with AMPKα1 and promoting UHRF1 degradation.

Taken together, these results suggested that BBR inhibited dephosphorylation to maintain AMPK activity, at least partially by promoting UHRF1 degradation and reducing its interaction with AMPKα1 (Figure 6).

## Discussion

As clinical drugs, both metformin and BBR activate AMPK to exert similar pharmacological activities. According to recent studies, metformin activated AMPK through PEN2 and AXIN1 in the lysosomal pathway at a clinically relevant dose, which was 5–300 μM depending on the OCT1 (metformin transporter) level on cell surface (Zhang et al., 2016; Ma et al., 2022).

In rats, 56% of BBR ran intact through the gastrointestinal tract after oral administration, and another 44% was disposed of by intestine first-pass elimination, leading to a low oral bioavailability of 0.36% (Liu et al., 2010). In human, the serum concentration was only 0.02 μM (6.99 ng/ml) for BBR (Yan et al., 2015). BBR concentrated in tissues like liver, heart, kidney, and lung after absorption (Liu et al., 2010). There was a 1.4–76 fold increase in the ratio of the area under the concentration-time curve value for BBR in these tissues than in plasma (Liu et al., 2010; Yan et al., 2015). Hence, 0.028–1.52 μM could be reachable in human tissues. And according to a previous report, 2.5 μM was used as the high dose for cell treatment (Zhang et al., 2014). Therefore, 0.1–2.5 μM was more clinically relevant doses for BBR, in different tissues.

We found that BBR activated lysosomal AMPK through AXIN1, but independent of PEN2. Metformin and BBR increased p-AMPK/total AMPK ratio, but not statistically significant in the gray scan result (Figure 1A). As Western blot was a semi-quantitative method, we performed ELISA detection shown in Figure 1B. The results show that the effect of metformin was significant (*p* = 0.0015), and BBR at 2.5 μM was close to the significant level (*p* = 0.0587). Hence, metformin was more potent than BBR on stimulating lysosomal AMPK. However, at the cellular level, the potency of 2.5 μM BBR was similar as 250 μM metformin. It is indicated that there might be other mechanisms for BBR to facilitate cellular AMPK activation, which was different from that of metformin acting primarily through the lysosomal pathway.

Then, we found that BBR was an AMPK activator reducing UHRF1 expression. It could be speculated that BBR maintained AMPK activity by downregulating UHRF1, which was reported to interact with AMPKα and facilitate PP2A dependent dephosphorylation (Xu et al., 2022). Hence, we conducted knockdown and knock-in approaches to prove the UHRF1 function on BBR-induced AMPK activation. Our results suggested BBR suppressed the dephosphorylation process of AMPKα, by reducing UHRF1 expression and its interaction with AMPKα1. To the best of our knowledge, the present study, for the first time, showed that BBR and metformin possessed different mechanisms on AMPK activation.

Our results show that overexpression of UHRF1, but not PPP2CA, was sufficient to inhibit the effect of BBR on stimulating AMPK. It is indicated that PP2A might be excessive in cells, and UHRF1 expression was more important on AMPK dephosphorylation. This is consistent with the previous report, in which UHRF1 suppresses AMPK activation by acting as a bridging factor between phosphatase PP2A and AMPK. UHRF1 overexpression dramatically enhanced the interaction between AMPKα and PPP2CA, but not other phosphatases. And PP2A suppresses AMPK activation in a UHRF1-dependent manner (Xu et al., 2022).

As reported, UHRF1 did not inhibit AMPK in lysosome, as the shuttling between cytoplasm and nucleus was prevented (Xu et al., 2022). In AXIN1^−/−^ cells, the formation of lysosomal AMPK-activating complex was abolished (Zhang et al., 2016), and thus AMPK could no longer anchor onto the surface of lysosome. Instead, most of AMPK might return to cytoplasm, and be bound by UHRF1 for further nuclear dephoshporylation. Similar situation may occur in AXIN1 siRNA transfected cells. AXIN1 downregulation would influence AMPK localization as well. Therefore, BBR did not activate AMPK in AXIN1^−/−^ and AXIN1 knockdown cells, although UHRF1 expression was downregulated (Figures 2B,D). It seems that lysosomal AMPK activation was prior to UHRF1 inhibition for BBR’s action.

In summary, BBR moderately activated lysosomal AMPK independent of PEN2, and maintained cellular AMPK activity by reducing UHRF1 expression and its interaction with AMPKα1, which was different from that of metformin.

## Data Availability

The original contributions presented in the study are included in the article/Supplementary Material, further inquiries can be directed to the corresponding authors.
