# Supplementary material for: Berberine stimulates lysosomal AMPK independent of PEN2 and maintains cellular AMPK activity through inhibiting the dephosphorylation regulator UHRF1
Source: Front Pharmacol. 2023 Apr 18;14:1148611. doi: 10.3389/fphar.2023.1148611 (PMC10151516; doi:10.3389/fphar.2023.1148611)
Supplement: Supplementary file 1 [file Image1.pdf]

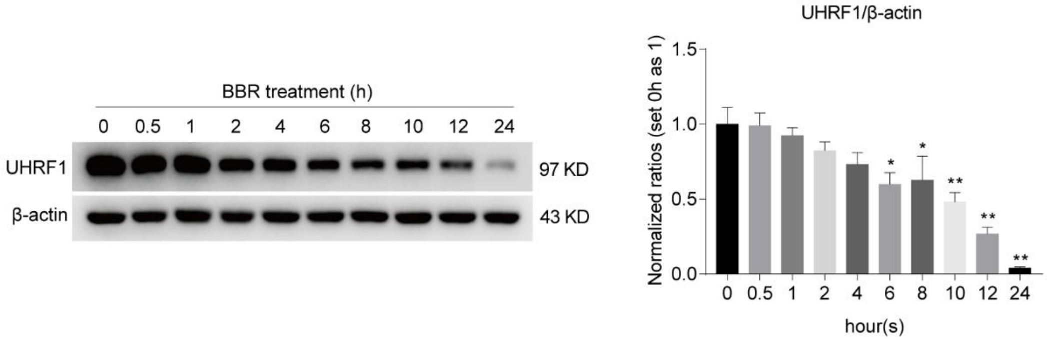

Supplementary Figure 1. BBR significantly reduced UHRF1 expression 6 h after cell treatment. HCT-116 cells were treated with BBR (2.5 μM) for 0, 0.5, 1, 2, 4, 6, 8, 10, 12 and 24 h, respectively. The ratio of 0 h was set as 1. $^{*}p < 0.05$, $^{**}p < 0.01$ versus that of the “0 h” group. Western blot was conducted as described in Methods.
